# Effects of Eyjafjallajökull Volcanic Ash on Innate Immune System Responses and Bacterial Growth *in Vitro*

**DOI:** 10.1289/ehp.1206004

**Published:** 2013-03-11

**Authors:** Martha M. Monick, Jonas Baltrusaitis, Linda S. Powers, Jennifer A. Borcherding, Juan C. Caraballo, Imali Mudunkotuwa, David W. Peate, Katherine Walters, Jay M. Thompson, Vicki H. Grassian, Gunnar Gudmundsson, Alejandro P. Comellas

**Affiliations:** 1 Department of Medicine, Carver College of Medicine, University of Iowa, Iowa City, Iowa, USA; 2 Department of Chemistry,; 3 Department of Geoscience, and; 4 Central Microscopy Research Facility, University of Iowa, Iowa City, Iowa, USA; 5 ARC Centre of Excellence in Ore Deposits, University of Tasmania, Hobart, Tasmania, Australia; 6 University of Iceland, Reykjavik, Iceland

**Keywords:** bacteria, epithelium, innate immunity, iron, macrophage, volcanic ash

## Abstract

Background: On 20 March 2010, the Icelandic volcano Eyjafjallajökull erupted for the first time in 190 years. Despite many epidemiological reports showing effects of volcanic ash on the respiratory system, there are limited data evaluating cellular mechanisms involved in the response to ash. Epidemiological studies have observed an increase in respiratory infections in subjects and populations exposed to volcanic eruptions.

Methods: We physicochemically characterized volcanic ash, finding various sizes of particles, as well as the presence of several transition metals, including iron. We examined the effect of Eyjafjallajökull ash on primary rat alveolar epithelial cells and human airway epithelial cells (20–100 µg/cm^2^), primary rat and human alveolar macrophages (5–20 µg/cm^2^), and *Pseudomonas aeruginosa* (PAO1) growth (3 µg/10^4^ bacteria).

Results: Volcanic ash had minimal effect on alveolar and airway epithelial cell integrity. In alveolar macrophages, volcanic ash disrupted pathogen-killing and inflammatory responses. In *in vitro* bacterial growth models, volcanic ash increased bacterial replication and decreased bacterial killing by antimicrobial peptides.

Conclusions: These results provide potential biological plausibility for epidemiological data that show an association between air pollution exposure and the development of respiratory infections. These data suggest that volcanic ash exposure, while not seriously compromising lung cell function, may be able to impair innate immunity responses in exposed individuals.

On 20 March 2010, Eyjafjallajökull, a volcano in Iceland, erupted for the first time in 190 years; this eruption was from a vent on the volcano’s flank (Swindles et al. 2011). A second and larger eruption occurred on 14 April from the summit; this eruption continued until late May 2011 (Sigmundsson et al. 2010). An interaction between ash and magma led to large volumes of finely comminuted ash entering the atmosphere (Gudmundsson 2011). The eruption plume was approximately 6 miles high, leading to significant ash dispersal to both Iceland and parts of Europe (Gislason et al. 2011). The average ash concentration in the cloud that reached Europe has been calculated at 10–35 mg/m^3^, with peak ambient air concentrations of particulate matter as high as 13 mg/m^3^ (Gudmundsson et al. 2012). Even after the eruption ceased, ambient air concentrations of ash were close to 1 mg/m^3^ (Thorsteinsson 2012). During the initial explosive phase on 14–16 April, approximately 35% of particles were < 30 mm in diameter (Gudmundsson et al. 2012). Ash collected immediately after the eruption contained up to 25% respirable particles (< 10 mm in diameter) (Gudmundsson 2011). In addition to particles, the gaseous component from eruptions can include sulfur dioxide and other species that may harm humans who are in proximity to the eruption (Schmidt et al. 2011).

Although volcanic eruptions are a fairly rare event, there is significant data supporting the adverse health effects of respirable particles. Lave and Seskin (1972) linked air pollution and premature mortality. This association has been confirmed in subsequent studies (Dockery et al. 1993; Elliott et al. 2007; Samet et al. 2000; Spix et al. 1993). Volcanic eruptions—because of the significant particle burden in the atmosphere—have adverse effects on human health, including bronchitis, asthma exacerbations, and respiratory symptoms leading to hospital admissions (Baxter 1983; Baxter et al. 1981).

During the 1980 Mount St. Helens eruption in Washington State (USA), several people died from asphyxia by volcanic ash and from thermal burns with airway injury (Bernstein et al. 1986). Subsequent studies examined the subacute and chronic effects of the eruption. Although asthma exacerbations, upper respiratory infections, otitis, and bronchitis were documented (Bernstein et al. 1986), some studies found only limited risk of lung infections (Martin et al. 1986). Regarding the Icelandic eruption, Carlsen et al. (2012) reported that Icelanders exposed to Eyjafjallajökull volcanic ash had increased prevalence of respiratory symptoms, specifically asthma and chronic bronchitis, compared with a control population in northern Iceland. Although other studies have shown that exposure to volcanic ash increases the risk of developing respiratory infections (Convit et al. 2006; Gudmundsson 2011; Naumova et al. 2007), there are limited data on the cellular mechanisms involved in this increased lung infection risk. Because airway infections are the result of impaired innate immune mechanisms, we hypothesized that volcanic ash impairs innate immune mechanisms, specifically the function of macrophages and antimicrobial peptides.

## Materials and Methods

*Volcanic ash collection and characterization*. Three batches of ash were collected shortly after the Eyjafjallajökull eruption in 2010. Ash A was collected by rescue team member B. Hardottir on 5 May 2010 at Vik village, about 38 km from the source, and sent to the Institute of Earth Sciences University of Iceland (Reykjavik, Iceland) for measurement of fluorine and grain size. Ash B was collected by S. Gislason, geologist at the Institute of Earth Sciences, on 15 April 2010; this sample was collected about 58 km from the source as ash fell. Ash C was collected on 3 May 2010 about 64 km from the source by P. Eggertsson, a farmer at Hraungerdi, and sent to the institute for measurement of fluorine and grain size. All experiments (including the sieved experiments) were performed using batch B. We used all three batches in the Western analyses of alveolar macrophages (autophagy markers and MAP kinases). In the other experiments, preliminary experiments showed similar responses in all three batches of ash. Therefore, we used ash B for most of the experiments because a larger amount was available.

Volcanic ash was characterized by scanning electron microscopy (SEM), X-ray photoelectron spectroscopy (XPS), and inductively coupled plasma mass spectrometry (ICP-MS) [see Supplemental Material, Figure S1 and Tables S1 and S2 (http://dx.doi.org/10.1289/ehp.1206004)].

ICP-MS. Samples were analyzed for elemental concentrations using a Thermo X-Series II quadrupole ICP-MS instrument (Thermo Fisher Scientific Inc., Waltham, MA, USA), following the methods of Peate et al. (2010). Ash samples were dissolved using a standard hydrofluoric acid (HF)–nitric acid (HNO_3_) digestion method.

Leaching experiments were carried out in 0.001 M ultrapure HNO_3_ using an ash to acid ratio of 1:25 (0.1 g ash to 2.5 mL water/acid), following the recommendations of Witham et al. (2005). The ash–leach mixtures were agitated by shaking for 90 min in a sealed polyethylene bottle in an ultrasonic bath. Leachates were filtered through a 0.45-µm cellulose acetate Millipore filter (Millipore Corporation, Billerica, MA, USA) prior to analysis. Data were corrected for blanks and instrumental drift and calibrated relative to a series of multielement solutions (1 ppb, 10 ppb, and 50 ppb) gravimetrically prepared using ultrapure Milli-Q water from a 1,000-ppm stock solution (Inorganic Ventures). We assessed accuracy by comparing analyses of two natural water standard reference materials [NIST 1640a (trace elements in natural water; National Institute of Standards and Technology, Gaithersburg, MD, USA) and SLRS-5 (river water reference material for trace metals; National Research Council of Canada, Ottawa, Ontario, Canada)] analyzed as unknown samples with their certified values. Final leaching data are presented as elemental concentrations in milligrams per kilogram of ash. The complete ICP-MS data set, including standards, is provided in Supplemental Material, Tables S1 and S2 (http://dx.doi.org/10.1289/ehp.1206004).

XPS. We used a custom-designed Kratos Axis Ultra XPS system (Kratos Analytical, Chestnut Ridge, NY, USA) as previously described by Baltrusaitis et al. (2007). Briefly, high-resolution spectra were acquired in the region of interest using the experimental parameters as described by Amornpitoksuk et al. (2012). Samples containing particles were mounted on indium foil. We used CasaXPS software (Casa Software Ltd.; http://www.casaxps.com/) to process the XPS data. We subtracted a Shirley-type background from each spectrum to account for inelastically scattered electrons that could contribute to the background (Baltrusaitis et al. 2011). Transmission-corrected relative sensitivity factor values from the Kratos library (http://www.casaxps.com/kratos/) were used for elemental quantification in CasaXPS.

*Particle preparation for* in vitro *experiments*. For *in vitro* experiments, sieved ash (20 µM) from the Eyjafjallajökull eruption was suspended in the appropriate cell culture media plus dipalmitoylphosphatidylcholine (10 µg/mL). Particle suspensions were sonicated for 20 sec immediately before being added to cell cultures. In some cases, aluminum oxide (Al_2_O_3_) particles were also used for cell exposures and were prepared in the same manner.

*Cell models*. Cells used in these experiments included primary human and rat alveolar macrophages, primary rat alveolar epithelial cells, and primary human airway epithelial cells. All procedures and protocols complied with applicable U.S. and/or international regulations (including institutional review board approval). All human participants gave written informed consent prior to the study. All animals were treated humanely and with regard for alleviation of suffering.

Human alveolar macrophages were obtained from recruited healthy nonsmoking subjects (age 20–40, equally divided between males and females). Bronchoalveolar lavage (BAL) was performed by instilling 20 mL of normal saline into a tertiary bronchus up to five times in three different lung segments. Slides were microscopically examined to ensure that > 95% of the cells were macrophages (Monick et al. 2006, 2008, 2010).

Human airway epithelial cells were obtained from the University of Iowa cell culture core and were seeded as described previously by Karp et al. (2002).

Rat alveolar macrophages were isolated from pathogen-free male Sprague-Dawley rats (Harlan Laboratories, Madison, WI, USA) weighing 200–225 g. Animals were housed under standard conditions (50% humidity and 12-hr dark/light cycle) at the University of Iowa animal facility. Fresh water and food was available *ad libitum*. The alveolar macrophages were isolated and purified by differential adherence to IgG-coated dishes. BAL fluid was spun and cell pellet was resuspended in antibiotic-free Dulbecco’s modified Eagle’s medium (DMEM) containing 10% fetal bovine serum (FBS). Cells were plated and allowed to adhere for 1 hr before experiments began.

Primary rat alveolar type II epithelial cells were isolated from male Sprague-Dawley rats as previously described (Dobbs et al. 1986). Briefly, the lungs were perfused via the pulmonary artery, lavaged, and digested with elastase (30 U/mL; Worthington Biochemical, Lakewood, NJ, USA) for 20 min at 37°C. The ATII cells were purified by differential adherence to IgG-coated dishes. We assessed number and viability of alveolar epithelial cells by trypan blue exclusion.

*Culture conditions*. Macrophages were cultured at 1 million/mL in standard tissue culture flasks (60 mM and 100 mM) in RPMI 1640 medium with gentamycin. Epithelial cells were plated in DMEM containing 10% FBS, 2 mM l-glutamine, 100 U/mL penicillin, 100 µg/mL streptomycin, and 2.5 µg/mL amphotericin. Human airway epithelial cells and rat alveolar epithelial cells were placed on filters in order to develop cell polarity. Experiments were performed 3 days after isolation.

*Volcanic ash particle concentrations*. All macrophages and epithelial cells were exposed to particles as a function of surface area (micrograms per square centimeter). Bacterial exposure was standardized to micrograms of ash particles per number of bacteria [micrograms per 10^4^ *Pseudomonas aeruginosa* (PAO1; obtained from J. Zabner, University of Iowa, Iowa City, IA, USA)].

*Scanning electron microscopy (SEM)*. Isolated rat or human macrophages exposed to volcanic ash for 2 hr were fixed overnight with 2.5% glutaraldehyde (Sabatini et al. 1963) in 0.1 M cacodylate buffer. After standard processing, the samples were mounted onto stubs and sputter-coated with a gold-palladium mixture (Karnovsky 1971; Monick et al. 2010). Imaging was performed with a Hitachi S-4800 field emission scanning electron microscope (Hitachi High Technologies America Inc., Schaumberg, IL).

*Transmission electron microscopy (TEM) and scanning transmission electron microscopy (STEM)/high-angle annular dark field (HAADF).* TEM and energy-dispersive spectroscopy (EDS) were performed using a JEOL 2100F transmission electron microscope (JEOL, Peabody, MA, USA) operating in scanning mode (STEM) equipped with a Gatan HAADF detector (Gatan, Pleasanton, CA, USA) and a Thermo EDS detector (Thermo, West Palm Beach, FL, USA). We used 200 kV accelerating voltage and a 0.7-nm probe in all experiments. Tissue sections (90 nm) were cut using a microtome equipped with a diamond knife.

*Western blot analysis*. We used the following antibodies for Western blot analysis: LC3 (autophagy marker light chain 3; catalog no. 2775), ubiquitin (catalog no. 3936), phosphorylated (phospho)-ERK (extracellular signal-regulated kinase; catalog no. 9101), and phospho-p38 (catalog no. 9215) from Cell Signaling Technology Inc. (Danvers, MA, USA); phospho-JNK (*c*-Jun N-terminal kinase; catalog no. 559309) from EMD Millipore (Billerica, MA, USA); and β-actin (catalog no. ab8226) from Abcam (Cambridge, MA). Whole-cell protein was obtained by lysing the cells on ice for 20 min in 200 μL of lysis buffer (0.05 M Tris, pH 7.4, 0.15 M NaCl, 1% NP-40), with added protease and phosphatase inhibitors [1 protease minitab (Roche Biochemicals, Indianapolis, IN, USA)/10 mL, and 100 µL 100× phosphatase inhibitor cocktail (Calbiochem, La Jolla, CA, USA)/10 mL]. The lysates were sonicated for 20 sec, kept at 4°C for 30 min, spun at 15,000 × *g* for 10 min, and the supernatant saved. Protein determinations were made using the Bradford Protein assay from Bio-Rad (Hercules, CA, USA). Cell lysates were stored at –70°C until use. For Western analysis, protein samples (30 µg whole-cell proteins) were mixed 1:1 with 2× sample buffer (20% glycerol, 4% SDS, 10% β-mercaptoethanol, 0.05% bromophenol blue, and 1.25 M Tris, pH 6.8; all from Sigma Chemical Co., St. Louis, MO, USA), heated to 95°C for 5 min, loaded onto a 10% SDS-PAGE gel, and run at 100 V for 90 min. Cell proteins were transferred to polyvinylidene fluoride (PVDF; Bio-Rad) by semi-dry transfer (BioRad). Equal loading of the protein groups on the blots was evaluated using Ponceaus S, designed for staining proteins on PVDF membranes or by stripping and reprobing with antibodies to β-actin. The PVDF was dried and incubated overnight with primary antibody in 5% milk. The blots were washed 4 times with TTBS and incubated for 1 hr with horseradish-peroxidase conjugated anti-rabbit or anti-mouse IgG. Immunoreactive bands were developed using a chemiluminescent substrate (ECL Plus, Amersham, Arlington Heights, IL, USA). Autoradiographs were developed for 10 sec to 2 min.

*Quantitative real-time reverse-transcriptase polymerase chain reaction (qRT-PCR) for TNF*α. RNA was isolated from human alveolar macrophages using reagents from the MirVana kit (Applied Biosystems, Austin, TX, USA) according to the manufacturer’s instructions. RNA quality and quantity were assessed with an Experion Automated Electrophoresis System (Bio-Rad) according to the manufacturer’s protocol. RNA quality was considered adequate for use if the 28S/18S ratio was > 1.2 and the RNA quality indicator was > 7. Total RNA (1 μg) was reverse-transcribed to cDNA using the iScript cDNA Synthesis Kit (Bio-Rad) following the manufacturer’s instructions. PCR reactions used 2 μL cDNA and 48 μL master mix, containing iQ SYBR Green Supermix (Bio-Rad), 15 pmol of forward primer, and 15 pmol of reverse primer, and were performed in a CFX96 Real-Time PCR Detection System (Bio-Rad) as follows: 3 min at 95°C, followed by 40 cycles of 10 sec at 95°C and 30 sec at 55°C. The fluorescence signal generated with SYBR Green I DNA dye was measured during annealing steps. Specificity of the amplification was confirmed using a melting curve analysis. Data were collected and recorded by CFX Manager Software (Bio-Rad) and expressed as a function of threshold cycle (CT). The relative quantity of the *TNF*α mRNA was normalized to the relative quantity of hypoxanthine phosphoribosyltransferase (HPRT), and the sample mRNA abundance was calculated by the 2^–ΔΔC_T_^ method. Gene-specific primers were custom-synthesized and purchased from Integrated DNA Technologies (Iowa City, IA, USA) based on design using gene-specific nucleotide sequences from the National Center for Biotechnology Information sequence databases (TNFα primers: forward, aggacaccatgagcactgaaagca; reverse, ttgagggtttgctacaacatgggc).

*Cell death measurements*. We evaluated cell death using propidium iodide (10 µg/mL phosphate-buffered saline) or trypan blue exclusion (Reisetter et al. 2011). Images were analyzed using ImageJ software (Rasband 2012).

*Transepithelial electrical conductance (G_t_) measurement*. We measured transepithelial electrical resistance in both rat and human cells using the Millicell Electrical Resistance System (Millipore Corporation, Bedford, MA, USA); G_t_ was calculated as its reciprocal.

*Bacterial growth assays*. PAO1 was grown overnight in M9 media (1 × M9 salts, 2.2 mM glucose, 0.002 M magnesium sulfate, 0.001 M calcium chloride, and 25 mM sodium succinate) and then exposed to 10 µg/mL iron(III) chloride [FeCl_3_; a soluble source of iron (Fe)], Al_2_O_3_ (10 µg/mL) to control for particle effects, or volcanic ash (10 µg/mL) for 9 hr at 37°C. We measured optical density at 600 nm (OD_600_), adjusting for particle absorbance effects, and recorded growth over the 9-hr period. Data were compared for all parameters of the growth curve by the extra-sum-of-squares *F*-test.

*Macrophage bacterial killing assay*. Isolated rat or human macrophages were exposed to 25 µg/mL volcanic ash for 2 hr and primed with 10 ng/mL lipopolysaccharide (LPS; LIST Biological Laboratories, Campbell, CA, USA) for 1 hr. Cells were then exposed to PAO1 [2.5 × 10^6^ colony forming units (CFU)]. At either 20 min or 110 min, cells were washed with 4 × Hanks’ balanced salt solution and harvested. Cells were lysed in ice-cold double-distilled water, samples were plated, and PAO1 CFUs were counted by visual inspection using a colony counter. To determine the effect of bacterial phagocytosis (at 20 min) and bacterial killing (at 110 min), we compared the number of PAO1 CFUs in ash-exposed macrophages with those of controls.

*Antimicrobial peptide activity*. PAO1, grown overnight in M9 media, was subcultured and diluted in M9 media to an OD_600_ of 0.45. The culture was then diluted 1/1,000, and a 10-µL aliquot was used for each experiment. A cocktail of antimicrobial peptides (600 µg/mL lysozyme, 200 µg/mL lactoferrin, and 100 ng/mL β-defensins 1 and 2) in sodium phosphate buffer (NaPi), pH 7.8 (a total of 400 µL) was added to a 96 deep-well plate. Volcanic ash (10 µg/mL), FeCl_3_ (10 µg/mL; positive control), Al_2_O_3_ (10 µg/mL), or media alone (control) was added to the plates containing antimicrobial peptides and PAO1, and the mixture was incubated for 1 hr at 37°C and 300 rpm. Luria broth (25%) was added to the mixture and grown overnight. The OD_600_ was measured to determine the level of antimicrobial peptide activity. To determine the number of CFUs, we conducted the experiment as described above, except the cultures were serially diluted and plated on Luria broth agar plates at the beginning and end of the experiment.

*Statistical analysis*. We used the unpaired Student’s *t*-test and one-way analysis of variance to determine significance between experimental groups. Data are presented as mean ± SE. Data analysis was performed using GraphPad Prism 5.00 (GraphPad Software, San Diego, CA, USA).

## Results

*Characterization of volcanic ash*. Ash B was used to characterize the volcanic ash from the Eyjafjallajökull eruption. SEM images of volcanic ash particles sieved through a < 20-µm sieve [see Supplemental Material, Figure S1A (http://dx.doi.org/10.1289/ehp.1206004)] show a distribution of particle sizes of two distinct fractions, 20 µm and < 2 µm. XPS analysis to determine the surface elemental composition of ash particles (see Supplemental Material, Table S1) indicated that volcanic ash is composed of alumosilicates with detectable concentrations of biologically relevant metals such as Fe. Metals, such as Fe and titanium, were present in localized areas (see Supplemental Material, Figure S1B). We performed ICP-MS to analyze elemental concentrations (in milligrams per killigram ash) in both the unsieved ash sample and the < 20-µm sieved fraction (see Supplemental Material, Table S2). Results of our ICP-MS compositional analyses of three separate unsieved ash samples are in excellent agreement with mean values reported previously (Borisova et al. 2012; Sigmarsson et al. 2011). Leaching experiments were also performed to better understand the propensity of the Icelandic volcanic ash toward dissolution in aqueous environments. Leaching the bulk ash in water released Fe at about 2 mg/kg ash, and leaching the bulk ash in weak acid released about 33 mg/kg ash. However, leaching the < 20-µm fraction in weak acid released Fe at 900 mg/kg ash (see Supplemental Material, Table S2).

**Figure 1:**
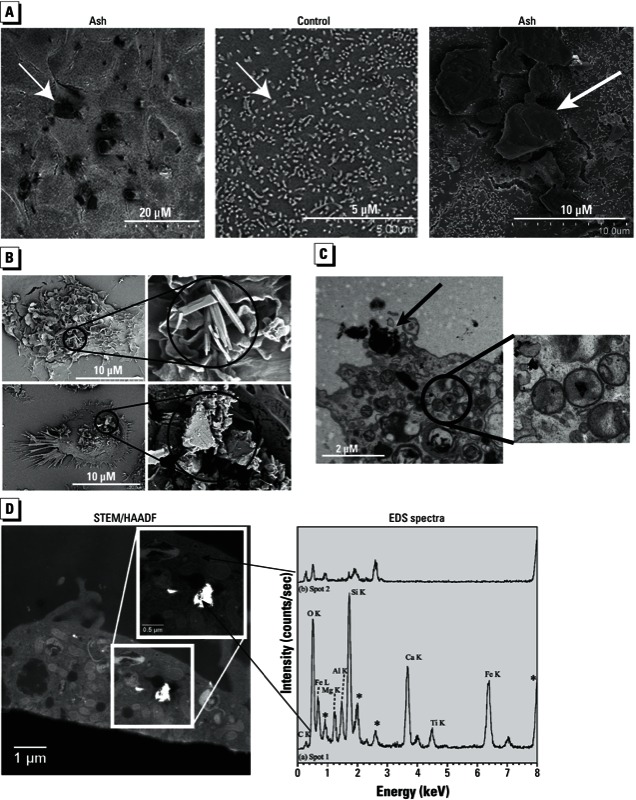
SEM of airway epithelial cells and alveolar macrophages exposed to volcanic ash for 3 hr. (*A*) Images of rat alveolar epithelial cells exposed to 20 µg/cm^2^ volcanic ash (left and right) or to standard media alone (control; center); white arrows point to micro­villi on cells. (*B*) Images (left) showing internalized volcanic ash in human alveolar macrophages exposed to 20 µg/cm^2^ volcanic ash; the indicated areas are shown at a higher magnification (right). TEM (*C*) and STEM/HAADF and EDS (*D*) analysis of human alveolar macrophages exposed to volcanic ash (2 µg/cm^2^). (*C*) Representative TEM image, with the circled area shown at a higher magnification (right); note the ash particles (black arrow) inside vesicles in the cytosol. (*D*) Representative STEM/HAADF image (left), with the indicated area shown at a higher magnification (inset); elemental analysis by EDS (right) shows elements found in particles, which are typical for those of volcanic ash. Asterisks indicate osmium peaks from thin-section staining.

*Interaction of volcanic ash with epithelial cells and macrophages*. In rat alveolar epithelial cells exposed to volcanic ash in culture, we observed ash particles on the cell surface (Figure 1A). In human alveolar macrophages exposed to volcanic ash, ash particles adhered to the surface (Figure 1B) or were internalized by alveolar macrophages and vesicles within the cell (Figure 1C). STEM/EDS elemental analysis of particles inside alveolar macrophages showed that these particles were rich in elements typical of those in volcanic ash (Figure 1D).

**Figure 2:**
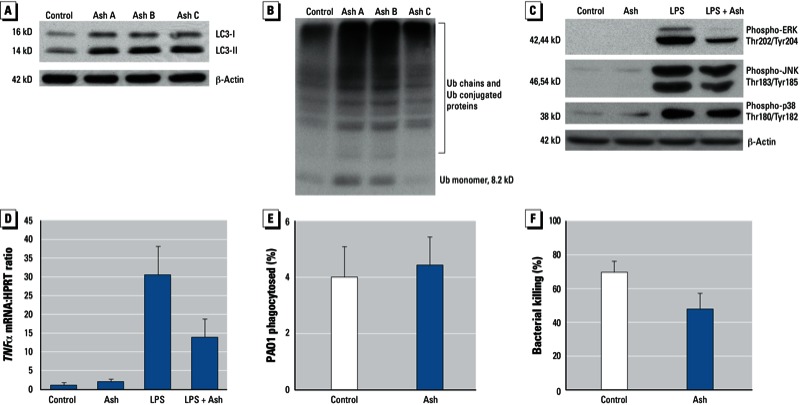
Effects of volcanic ash exposure on macrophage function. (*A,B*) Results of Western blot analysis of whole-cell proteins from human alveolar macro­phages cultured for 5 hr with media alone (control) or with ash (20 µg/cm^2^) from batch A, B, or C. (*A*) LC3‑II levels indicate an accumulation of auto­phagosomes. (*B*) Increased ubiquitin (Ub) conjugates are present at all molecular weights. (*C*) Western blot analysis of human alveolar macrophages exposed to ash (batch B; 20 µg/cm^2^) for 30 min and then to LPS (100 ng/mL) for 30 min; Phosphorylated (activated) MAP kinases decrease in LPS activation with ash exposure. Ash exposure reduced LPS activation of phospho-ERK, phospho-JNK, and phospho-p38. For (*A–C*), blots shown are representative of three experiments. (*D*) Real-time RT-PCR analysis showing *TNF*α mRNA in human alveolar macrophages exposed to ash (20 µg/cm^2^) for 30 min and then exposed to LPS (100 ng/mL) for 3 hr; data represent mean ± SE of three experiments. (*E,F*) Phagocytosis (*E*) and bacterial killing (*F*) in rat alveolar macrophages exposed to media alone (control) or to 25 µg/mL ash for 2 hr, primed with 10 ng/mL LPS for 1 hr, and then exposed to PAO1 (2.5 × 10^6^ CFU) for 20 min (phagocytosis) or 110 min (bacterial killing); data shown are mean ± SE of four experiments in triplicate. (*E*) Phagocytosis was similar in control and ash-exposed macrophages. (*F*) Bacterial killing was inhibited in ash-exposed macrophages, although not statistically significantly (*p* = 0.07).

*Effects of volcanic ash on cell function*. Autophagy is an important cellular homeostatic mechanism that clears particulates, protein aggregates, old and damaged mitochondria, and cytosolic bacteria (Gutierrez et al. 2004; Monick et al. 2010). Thus, we examined the effect of ash on homeostatic mechanisms (autophagic vesicles and clearance of ubiquitin-tagged proteins) in human alveolar macrophages exposed to volcanic ash (batch A, B, or C; 20 µg/cm^2^) using Western blot analysis. Figure 2A shows increased levels of LC3-II with each ash exposure, suggesting that ash either increases generation of autophagosomes or blocks progression of autophagosomes to fuse with lysosomes. To determine which of these was occurring, we analyzed total ubiquitin levels by Western blot analysis. We observed an accumulation of ubiquitin-conjugated proteins in ash-exposed cells (Figure 2B), suggesting that ash exposure may interfere with autophagy in human alveolar macrophages.

All three MAP kinase pathways (ERK, JNK, and p38) are required for optimal cytokine responses in these cells (Carter et al. 1999a, 1999b; Monick et al. 1999). Therefore, we measured activation of MAP kinase pathways and cytokine production after LPS exposure to examine the effect of ash exposure on inflammatory pathways. Figure 2C shows LPS-induced activation of ERK, p38, and JNK (indicated by an increase in phosphorylation of the activated tyrosine and threonine sites) that was inhibited by ash exposure. Ash exposure led to decreased activation of JNK and ERK by LPS but had no effect on p38 activity. Because LPS-induced ERK and JNK activity was inhibited, we tested the effect of ash exposure on *TNF*α mRNA expression after LPS. Figure 2D shows decreased production of *TNF*α mRNA by LPS in human alveolar macrophages exposed to ash.

We examined whether volcanic ash impairs the ability of macrophages to kill bacteria. When we tested phagocytosis of bacteria after a preincubation with ash, we found no difference in bacterial uptake between control and ash-exposed rat alveolar macrophages (Figure 2E). In the bacterial killing assay, we observed a trend toward fewer bacteria in the ash-exposed cells than in the control cells (*p* = 0.07) (Figure 2F). This result was concentration dependent: Exposure of macrophages to ash at 5 µg/cm^2^ impaired the cells’ ability to kill bacteria, whereas the response to a concentration of 2 µg/cm^2^ was no different from controls (data not shown).

**Figure 3:**
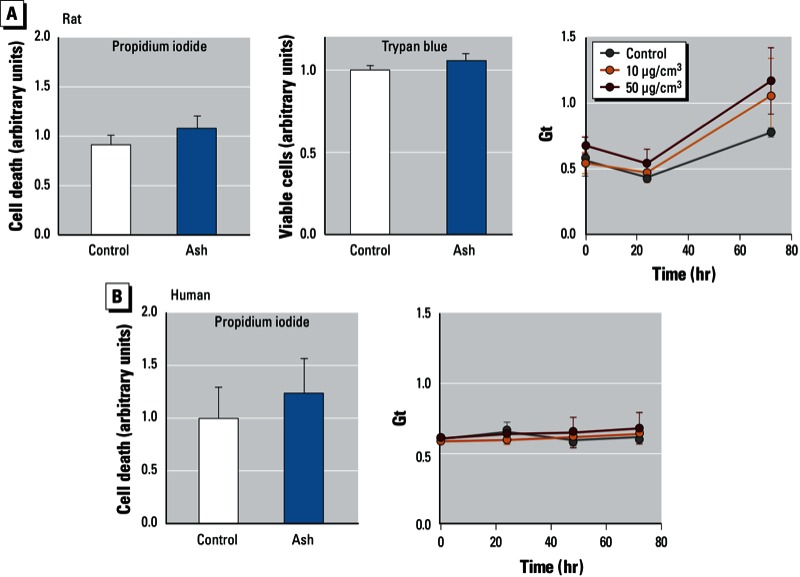
Electrical resistance and viability of rat alveolar epithelial cells (*A*) and human airway epithelial cells (*B*) exposed to 10, 50, or 100 µg/cm^2^ of sieved volcanic ash. Cell death evaluated by propidium iodide staining or trypan blue exclusion is shown for control cells and ash-exposed (100 µg/cm^2^) cells (*n* = 3 in triplicate for rat cells; *n* = 2 in triplicate for human cells). Transepithelial conductance (G_t_) is shown for control and ash-exposed (10 and 50 µg/cm^2^) cells (*n* = 3 in triplicate for both cell types). Data represent mean ± SE. Neither cell death nor G_t_ was significantly increased by ash exposure in either cell type.

We examined whether volcanic ash would induce cell death (propidium iodide or trypan blue exclusion) or epithelial barrier disruption (transepithelial electrical resistance) in human or rat epithelial cells. Volcanic ash did not induce cell death in either rat or human epithelial cells (Figure 3). Although particulate matter has been reported to induce disruption of epithelial barrier integrity (Caraballo et al. 2011b; Petecchia et al. 2009; Slebos et al. 2007; Soberanes et al. 2009; Upadhyay et al. 2003), we observed that epithelial cell barrier integrity was preserved in the presence of volcanic ash in both rat and human cells (Figure 3). Our results indicate that Eyjafjallajökull volcanic ash does not induce alveolar epithelial injury, even at high concentrations. This is different from studies in our laboratory on other particulates, in which both ambient air particles and diesel exhaust particles were found to increase transepithelial conductance (Caraballo et al. 2011b, 2012).

**Figure 4:**
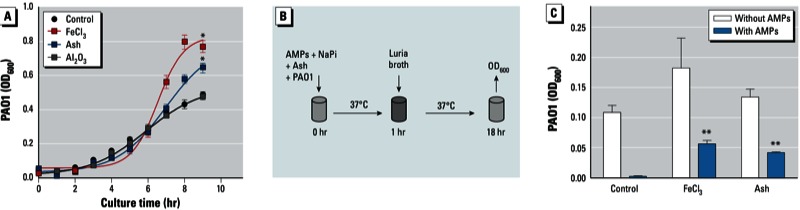
Effect of volcanic ash on bacterial growth and on bacterial killing by antimicrobial peptides (AMPs). (*A*) Growth of PAO1 after 9 hr incubation with media alone (control), FeCl_3_ (10 µg/mL; positive control), ash (10 µg/mL), or Al_2_O_3_ (control for particle effects; 10 µg/mL) as measured at OD_600_. PAO1 growth was increased after ash exposure compared with the control. (*B*) Schematic of the AMP activity assay. (*C*) Results of the AMP activity assay showing the bacterial killing capacity as determined by OD_600_ measurement. For (*A*) and (*C*), *n* = 3 in triplicate. *p < 0.0001 compared with the control by Student’s *t*-test. **p < 0.0001 compared with FeCl_3_ by Student’s *t*-test.

*The effect of volcanic ash on bacterial growth and killing capacity of antimicrobial peptides.* We examined whether volcanic ash increased bacteria growth by adding 3 µg of sieved volcanic ash particles to 3-hr subcultured PAO1 (10^4^). Volcanic ash can release readily soluble Fe into the environment (Olgun et al. 2011), and the volcanic ash from Eyjafjallajökull is rich in Fe (~ 75,000 mg/kg ash) [see Supplemental Material, Table S2 (http://dx.doi.org/10.1289/ehp.1206004)].Therefore, we used FeCl_3_ (25 µm), a soluble Fe source, as a positive control. As shown in Figure 4A, bacterial growth was significantly increased after ash exposure compared with the control and the Fe-deficient Al_2_O_3_ (*p* < 0.0001). This result suggests that volcanic ash can be a bioavailable source of Fe for PAO1 growth.

In the lungs, antimicrobial peptides are located in airway surface liquid (ASL), which primarily contains lactoferrin, lysozyme, and β-defensins 1 and 2. Lysozyme degrades the bacterial cell wall, β-defensins have broad antibacterial activity, and lactoferrin sequesters Fe and inhibits microbial respiration (Borcherding et al. 2013; Wiesner and Vilcinskas 2010). To test whether volcanic ash inhibits antimicrobial peptide activity, we combined physiologically relevant concentrations of antimicrobial peptides with volcanic ash (3 µg ash per 10^4^ PAO1) or FeCl_3_ and observed the effect on PAO1 growth (Figure 4B). In the presence of antimicrobial peptides, PAO1 did not grow (Figure 4C), indicating killing of bacteria by the antimicrobial peptide cocktail. However, when ash was added, the inhibitory effect of antimicrobial peptides on PAO1 growth was compromised (Figure 4C; *p* ≤ 0.0001). Thus, in this experiment, volcanic ash inhibited antimicrobial peptide activity.

## Discussion

Approximately 10% of the world population lives within 100 km of historically active volcanos. Of all the potential hazards of an eruption, ash may have the widest impact on human health. This is because volcanic ash is respirable, can be transported to distal sites by the wind, and can remain in the environment for long periods of time. Although eruptions are often short-lived, ash fall deposits can remain in the local environment for years to decades, being remobilized by human activity or simply resuspended by wind (Hansell et al. 2006).

Populations exposed to volcanic air pollution have been reported to have increased prevalence and incidence of upper and lower respiratory tract infections (Amaral and Rodrigues 2007; Longo and Yang 2008; Naumova et al. 2007). The Mount St. Helens eruption was one of the most studied eruptions in terms of potential health effects of volcanic activity. During the 2 weeks after the eruption, emergency room visits increased significantly in affected areas. The major reasons for emergency room visits were upper respiratory infections and otitis (Bernstein et al. 1986). Grose et al. (1985) reported that intratracheal instillation of both fine and coarse Mount St. Helens volcanic ash caused small but a significant increases in susceptibility of mice to streptococcal infections when the ash was instilled 24 hr before bacterial challenge. In a recent study, Carlsen et al. (2012) reported that Icelanders exposed to Eyjafjallajökull volcanic ash had increase prevalence of respiratory symptoms; however, the effects of volcanic ash on bacterial growth and innate immunity were not established.

Immediately after the Icelandic eruption, peak particulate concentrations were as high as 13 mg/m^3^, and after the eruption ceased, measurements were near 1 mg/m^3^ (Thorsteinsson 2012). Taking these two concentrations and assuming a minute ventilation of 6 L/min (~ 8.6 m^3^ over 24 hr) for a healthy adult at rest, the total dose inhaled over 24 hr would be 111.8 mg for the peak particulate concentration (13 mg/m^3^) and 8.6 mg for the 1-mg/m^3^ concentration. However, because it is difficult to predict whether an exposed individual may have used a nasal or oral–nasal breathing pattern and to predict the size distribution of particulates inhaled, it is safe to assume that only 50% of inhaled particles would be deposited in the lung. Assuming that the surface area of a human airway is 4,430 cm^2^, in the first day a healthy subject would accumulate particulates at approximately 126 µg/cm^2^ during the peak concentration and approximately 1 µg/cm^2^ when exposed to 1 mg/m^3^. In our experiments with airway epithelial cells, we used ash concentrations of 10, 50, and 100 µg/cm^2^. Therefore, the concentrations we used are within the range of potential human exposure concentrations on the days after the eruption. Ash collected immediately after the eruption contained up to 25% respirable particles (< 10 µm, the size of particles that reach the lung) (Gudmundsson 2011). Most of our experiments were performed with volcanic ash closer to the respirable particle fraction (< 20 µm).

Macrophage function is impaired by exposure to volcanic ash; that is, bactericidal activity is impaired but phagocytosis is not. It is likely that the ash overwhelms the capacity of macrophages to kill bacteria. At lower concentrations, ash inhibited antimicrobial peptide activity, thus impairing bactericidal and bacteriostatic activity of the airway. Because metal content is abundant in volcanic ash as well as in fly ash, our findings have implications for studies on the effects of fly ash and innate immunity. For example, Roberts et al. (2009) examined the effect of the soluble metal fraction of residual oil fly ash on immunity to *Listeria monocytogenes*. They found a link between nickel from fly ash and reduced lung immunity in a rat model (Roberts et al. 2009). In addition, Klein-Patel et al. (2006) showed that residual oil fly ash inhibits β-defensin gene expression in bovine and human lung epithelial cell lines.

The physicochemical characteristics of volcanic particulate matter will determine some health effects associated with exposures to ash from volcanic eruptions. In general, volcanic and other forms of particulates, such as coal fly ash and urban particulates, contain a number of metals, including Fe. Of the total Fe in volcanic ash, Fe^3+^ comprises only 10–15%, with the remainder being mainly Fe^2+^. Although Fe is predominantly in the Fe^2+^ oxidation state in volcanic materials (most likely in the form of silicate glasses, silicate minerals, and Fe–titanium oxides), it will be oxidized as it is released into the atmosphere; thus, the Fe will be present in the Fe^3+^ oxidation state in the leachates (Kelley and Cottrell 2009). Because free Fe levels are very low in biological fluids (< 10^–18^ M) (Bullen et al. 2005), particulates can be an exogenous Fe source for bacteria. Furthermore, Fe mobilization in coal fly ash is associated with aluminosilicate glass phases (Veranth et al. 2000), particle size (Chen et al. 2012), and Fe speciation (Fu et al. 2012). Therefore, knowledge of the total Fe content is not enough to explain or predict the propensity of Fe solubility in Fe-containing particles such as volcanic ash.

## Conclusions

**Figure 5:**
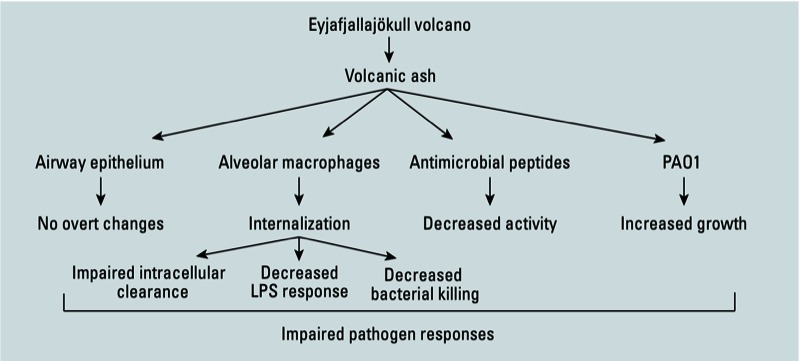
Diagram showing effects of Eyjafjallajökull volcanic ash on the innate immune system and on bacterial growth.

Results of this study suggest that exposure to respirable volcanic ash may increase the likelihood of developing bacterial infections via effects on both the bacteria and on the innate immune system (Figure 5). Our *in vitro* experiments showed that alveolar macrophage function and antibacterial peptide activity were compromised after exposure to ash from the Eyjafjallajökull volcano. In addition, we observed increased growth of volcanic ash–exposed *P. aeruginosa*. We suggest that these data provide a new mechanistic paradigm for the adverse effects of volcanic ash exposure on respiratory health.

## Supplemental Material

(438 KB) PDF

## References

[r1] Amaral AF, Rodrigues AS (2007). Chronic exposure to volcanic environments and chronic bronchitis incidence in the Azores, Portugal.. Environ Res.

[r2] Amornpitoksuk P, Suwanboon S, Sangkanu S, Sukhoom A, Muensit N, Baltrusaitis J (2012). Synthesis, characterization, photocatalytic and antibacterial activities of Ag-doped ZnO powders modified with a diblock copolymer.. Powder Technol.

[r3] Baltrusaitis J, Jayaweera PM, Grassian VH (2011). Sample pretreatment, and light on surface speciation and surface coverage.. J Phys Chem.

[r4] Baltrusaitis J, Usher CR, Grassian VH (2007). Reactions of sulfur dioxide on calcium carbonate single crystal and particle surfaces at the adsorbed water carbonate interface.. Phys Chem Chem Phys.

[r5] Baxter PJ (1983). Health hazards of volcanic eruptions.. J R Coll Physicians Lond.

[r6] Baxter PJ, Ing R, Falk H, French J, Stein GF, Bernstein RS (1981). Mount St Helens eruptions, May 18 to June 12, 1980. An overview of the acute health impact.. JAMA.

[r7] Bernstein RS, Baxter PJ, Falk H, Ing R, Foster L, Frost F (1986). Immediate public health concerns and actions in volcanic eruptions: lessons from the Mount St. Helens eruptions, May 18–October 18, 1980.. Am J Public Health.

[r8] Borcherding JA Chen H Caraballo JC Baltrusaitis J Pezzulo AA Zabner J 2013 Coal fly ash impairs airway antimicrobial peptides and increases bacterial growth. PLoS ONE 8 2 e57673; doi:10.1371/journal.pone.0057673 [Online 28 February 2013] 23469047 PMC3585163

[r9] Borisova AY Toutain JP Stefansson A Gouy S de Parseval P 2012 Processes controlling the 2010 Eyjafjallajökull explosive eruption. J Geophys Res Solid Earth 117 B05); doi:10.1029/2012JB009213 [Online 3 May 2012]

[r10] Bullen JJ, Rogers HJ, Spalding PB, Ward CG (2005). Iron and infection: the heart of the matter.. FEMS Immunol Med Microbiol.

[r11] Caraballo JC, Borcherding J, Thorne PS, Comellas AP (2012). Protein kinase C–ζ mediates diesel exhaust particles-induced lung injury.. Am J Respir Cell Mol Biol.

[r12] Caraballo JC, Yshii C, Butti ML, Westphal W, Borcherding JA, Allamargot C (2011a). Hypoxia increases transepithelial electrical conductance and reduces occludin at the plasma membrane in alveolar epithelial cells via PKC-ζ and PP2A pathway.. Am J Physiol Lung Cell Mol Physiol.

[r13] Caraballo JC, Yshii C, Westphal W, Moninger T, Comellas AP (2011b). Ambient particulate matter affects occludin distribution and increases alveolar transepithelial electrical conductance.. Respirology.

[r14] Carlsen HK Hauksdottir A Valdimarsdottir UA Gíslason T Einarsdottir G Runolfsson H 2012 Health effects following the Eyjafjallajökull volcanic eruption: a cohort study. BMJ Open 2 e001851; doi:10.1136/bmjopen-2012-001851 [Online 8 November 2012] PMC3533043 23144261

[r15] Carter AB, Knudtson KL, Monick MM, Hunninghake GW (1999a). The p38 mitogen-activated protein kinase is required for NF-κB-dependent gene expression. The role of TATA-binding protein (TBP).. J Biol Chem.

[r16] Carter AB, Monick MM, Hunninghake GW (1999b). Both Erk and p38 kinases are necessary for cytokine gene transcription.. Am J Respir Cell Mol Biol.

[r17] Chen H, Laskin A, Baltrusaitis J, Gorski CA, Scherer MM, Grassian VH (2012). Coal fly ash as a source of iron in atmospheric dust.. Environ Sci Technol.

[r18] Convit J, Ulrich M, Castillo J, De Lima H, Perez M, Caballero N (2006). Inorganic particles in the skin of inhabitants of volcanic areas of Central America: their possible immunomodulatory influence in leishmaniasis and leprosy.. Trans R Soc Trop Med Hyg.

[r19] Dobbs LG, Gonzalez R, Williams MC (1986). An improved method for isolating type II cells in high yield and purity.. Am Rev Respir Dis.

[r20] Dockery DW, Pope CA, Xu X, Spengler JD, Ware JH, Fay ME (1993). An association between air pollution and mortality in six U.S. Cities.. N Engl J Med.

[r21] Elliott P, Shaddick G, Wakefield JC, de Hoogh C, Briggs DJ (2007). Long-term associations of outdoor air pollution with mortality in Great Britain.. Thorax.

[r22] Fu H, Lin J, Shang G, Dong W, Grassian VH, Carmichael GR (2012). Solubility of iron from combustion source particles in acidic media linked to iron speciation.. Environ Sci Technol.

[r23] Gislason SR, Hassenkam T, Nedel S, Bovet N, Eiriksdottir ES, Alfredsson HA (2011). Characterization of Eyjafjallajökull volcanic ash particles and a protocol for rapid risk assessment.. Proc Natl Acad Sci USA.

[r24] Grose EC, Grady MA, Illing JW, Daniels MJ, Selgrade MK, Hatch GE (1985). Inhalation studies of Mt. St. Helens volcanic ash in animals. III. Host defense mechanisms.. Environ Res.

[r25] Gudmundsson G (2011). Respiratory health effects of volcanic ash with special reference to Iceland. A review.. Clin Respir J.

[r26] Gudmundsson MT Thordarson T Höskuldsson A Larsen G Björnsson H Prata FJ 2012 Ash generation and distribution from the April–May 2010 eruption of Eyjafjallajökull, Iceland. Sci Rep 2 572; doi:10.1038/srep00572 [Online 14 August 2012] 22893851 PMC3418519

[r27] Gutierrez MG, Master SS, Singh SB, Taylor GA, Colombo MI, Deretic V (2004). Autophagy is a defense mechanism inhibiting BCG and *Mycobacterium tuberculosis* survival in infected macrophages.. Cell.

[r28] Hansell AL, Horwell CJ, Oppenheimer C (2006). The health hazards of volcanoes and geothermal areas.. Occup Environ Med.

[r29] Karnovsky MJ (1971). Use of ferrocyanide reduced osmium tetroxide in electron microscopy [Abstract]. In: Abstracts of Papers, Eleventh Annual Meeting, the American Society for Cell Biology, the Jung Hotel, New Orleans, Louisiana, November 17–20, 1971.

[r30] Karp PH, Moninger TO, Weber SP, Nesselhauf TS, Launspach JL, Zabner J (2002). An in vitro model of differentiated human airway epithelia. Methods for establishing primary cultures.. Methods Mol Biol.

[r31] Kelley KA, Cottrell E (2009). Water and the oxidation state of subduction zone magmas.. Science.

[r32] Klein-Patel ME, Diamond G, Boniotto M, Saad S, Ryan LK (2006). Inhibition of beta-defensin gene expression in airway epithelial cells by low doses of residual oil fly ash is mediated by vanadium.. Toxicol Sci.

[r33] Lave LB, Seskin EP (1972). Air pollution, climate, and home heating: their effects on U.S. mortality rates.. Am J Public Health.

[r34] Longo BM, Yang W (2008). Acute bronchitis and volcanic air pollution: a community-based cohort study at Kilauea Volcano, Hawai`i, USA.. J Toxicol Environ Health A.

[r35] Martin TR, Wehner AP, Butler J (1986). Evaluation of physical health effects due to volcanic hazards: the use of experimental systems to estimate the pulmonary toxicity of volcanic ash.. Am J Public Health.

[r36] Monick MM, Carter AB, Gudmundsson G, Mallampalli R, Powers LS, Hunninghake GW (1999). A phosphatidylcholine-specific phospholipase C regulates activation of p42/44 mitogen-activated protein kinases in lipopolysaccharide-stimulated human alveolar macrophages.. J Immunol.

[r37] Monick MM, Powers LS, Barrett CW, Hinde S, Ashare A, Groskreutz DJ (2008). Constitutive ERK MAPK activity regulates macrophage ATP production and mitochondrial integrity.. J Immunol.

[r38] Monick MM, Powers LS, Gross TJ, Flaherty DM, Barrett CW, Hunninghake GW (2006). Active ERK contributes to protein translation by preventing JNK-dependent inhibition of protein phosphatase 1.. J Immunol.

[r39] Monick MM, Powers LS, Walters K, Lovan N, Zhang M, Gerke A (2010). Identification of an autophagy defect in smokers’ alveolar macrophages.. J Immunol.

[r40] Naumova EN Yepes H Griffiths JK Sempértegui F Khurana G Jagai JS 2007 Emergency room visits for respiratory conditions in children increased after Guagua Pichincha volcanic eruptions in April 2000 in Quito, Ecuador observational study: time series analysis. Environ Health 6 21; doi:10.1186/1476-069X-6-21 [Online 24 July 2007] 17650330 PMC1947976

[r41] Olgun N Duggen S Croot PL Delmelle P Dietze H Schacht U 2011 Surface ocean iron fertilization: the role of subduction zone and hotspot volcanic ash and fluxes into the Pacific Ocean. Global Biogeochemical Cycles 25 GB4001; doi:10.1029/2009GB003761

[r42] Peate DW, Breddam K, Baker JA, Kurz MD, Barker AK, Prestvik T (2010). Compositional characteristics and spatial distribution of enriched Icelandic mantle components.. J Petrol.

[r43] Petecchia L, Sabatini F, Varesio L, Camoirano A, Usai C, Pezzolo A (2009). Bronchial airway epithelial cell damage following exposure to cigarette smoke includes disassembly of tight junction components mediated by the extracellular signal-regulated kinase 1/2 pathway.. Chest.

[r44] Philibert RA, Sears RA, Powers LS, Nash E, Bair T, Gerke AK (2012). Coordinated DNA methylation and gene expression changes in smoker alveolar macrophages: specific effects on VEGF receptor 1 expression.. J Leukoc Biol.

[r45] Rasband WS (2008). ImageJ. Bethesda, MD:National Institutes of Health.. http://imagej.nih.gov/ij/.

[r46] Reisetter AC, Stebounova LV, Baltrusaitis J, Powers L, Gupta A, Grassian VH (2011). Induction of inflammasome-dependent pyroptosis by carbon black nanoparticles.. J Biol Chem.

[r47] Roberts JR, Young SH, Castranova V, Antonini JM (2009). The soluble nickel component of residual oil fly ash alters pulmonary host defense in rats.. J Immunotoxicol.

[r48] Sabatini DD, Bensch K, Barrnett RJ (1963). Cytochemistry and electron microscopy. The preservation of cellular ultrastructure and enzymatic activity by aldehyde fixation.. J Cell Biol.

[r49] Samet JM, Dominici F, Curriero FC, Coursac I, Zeger SL (2000). Fine particulate air pollution and mortality in 20 U.S. cities, 1987–1994.. N Engl J Med.

[r50] Schmidt A, Ostro B, Carslaw KS, Wilson M, Thordarson T, Mann GW (2011). Excess mortality in Europe following a future Laki-style Icelandic eruption.. Proc Natl Acad Sci USA.

[r51] Sigmarsson O Vlastelic I Andreasen R Bindeman I Devidal JL Moune S 2011 Remobilization of silicic intrusion by mafic magmas during the 2010 Eyjafjallajökull eruption. Solid Earth 2 271 281; doi:10.5194/se-2-271-2011 [Online 2 December 2011]

[r52] Sigmundsson F, Hreinsdóttir S, Hooper A, Arnadóttir T, Pedersen R, Roberts MJ (2010). Intrusion triggering of the 2010 Eyjafjallajökull explosive eruption.. Nature.

[r53] Slebos DJ, Ryter SW, van der Toorn M, Liu F, Guo F, Baty CJ (2007). Mitochondrial localization and function of heme oxygenase-1 in cigarette smoke-induced cell death.. Am J Respir Cell Mol Biol.

[r54] Soberanes S, Urich D, Baker CM, Burgess Z, Chiarella SE, Bell EL (2009). Mitochondrial complex III-generated oxidants activate ASK1 and JNK to induce alveolar epithelial cell death following exposure to particulate matter air pollution.. J Biol Chem.

[r55] Spix C, Heinrich J, Dockery D, Schwartz J, Volksch G, Schwinkowski K (1993). Air pollution and daily mortality in Erfurt, East Germany, 1980–1989.. Environ Health Perspect.

[r56] Swindles GT, Lawson IT, Savov IP, Connor CB, Plunkett G (2011). A 7000 yr perspective on volcanic ash clouds affecting northern Europe.. Geology.

[r57] Thorsteinsson T Jóhannsson T Stohl A Kristiansen NI 2012 High levels of particulate matter in Iceland due to direct ash emissions by the Eyjafjallajökull eruption and resuspension of deposited ash. J Geophys Res 117; doi:10.1029/2011JB008756 [Online 10 January 2012]

[r58] Upadhyay D, Panduri V, Ghio A, Kamp DW (2003). Particulate matter induces alveolar epithelial cell DNA damage and apoptosis: role of free radicals and the mitochondria.. Am J Respir Cell Mol Biol.

[r59] Veranth JM, Smith KR, Huggins F, Hu AA, Lighty JS, Aust AE (2000). Mossbauer spectroscopy indicates that iron in an aluminosilicate glass phase is the source of the bioavailable iron from coal fly ash.. Chem Res Toxicol.

[r60] Wiesner J, Vilcinskas A (2010). Antimicrobial peptides: the ancient arm of the human immune system.. Virulence.

[r61] Witham CS, Oppenheimer C, Horwell CJ (2005). Volcanic ash-leachates: a review and recommendations for sampling methods.. J Volcanol Geoth Res.

