# Supplementary material for: Effects of Eyjafjallajökull Volcanic Ash on Innate Immune System Responses and Bacterial Growth in Vitro
Source: Environ Health Perspect. 2013 Mar 11;121(6):691–8. doi: 10.1289/ehp.1206004 (PMC3672917; doi:10.1289/ehp.1206004)
Supplement: (438 KB) PDF [file ehp.1206004.s001.pdf]

Supplemental Material

Effects of Eyjafjallajökull Volcanic Ash on Innate Immune System Responses and Bacterial Growth *in Vitro*

Martha M. Monick[1], Jonas Baltrusaitis[2], Linda S. Powers[1], Jennifer A. Borcherding[1], Juan C. Caraballo[1], Imali Mudunkotuwa[2], David W. Peate[3], Katherine Walters[4], Jay M. Thompson[5], Vicki H. Grassian[2], Gunnar Gudmundsson[6], and Alejandro P. Comellas[1]

[1]Department of Medicine, Carver College of Medicine, University of Iowa, Iowa City, Iowa, USA

[2]Department of Chemistry, University of Iowa, Iowa City, Iowa, USA

[3]Department of Geoscience, University of Iowa, Iowa City, Iowa, USA

[4] Central Microscopy Research Facility, University of Iowa, Iowa City, Iowa, USA

[5]ARC Centre of Excellence in Ore Deposits, University of Tasmania, Hobart, Tasmania, Australia

[6]University of Iceland, Reykjavik, Iceland

Table of Contents

Supplemental Figure S1 page 2

Table S1 page 3

Table S2 page 4

Table S3 page 5

References page 6

Figure S1

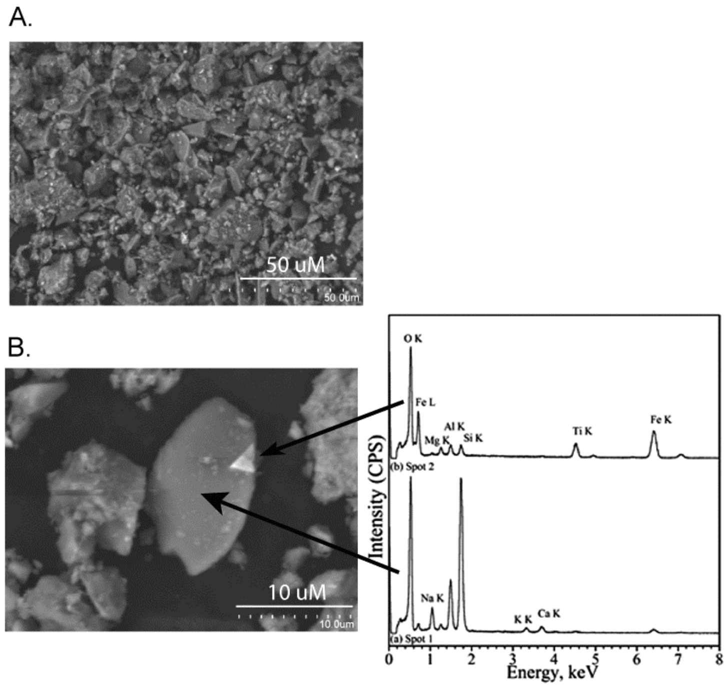

Figure S1.

Figure S1. A. Scanning electron microscopy (SEM) images of sieved volcanic ash particles (<20 µm) showing distribution of sizes. B. SEM image of a typical volcanic ash particle shows bright inclusion rich in Fe and Ti. SEM/EDS point spectra confirms particle analysis. The majority component was oxygen with 60.7±0.1 atomic % showing that particles are largely comprised of metal oxides and aluminosilicate clays. Titanium and iron were not uniformly distributed in the particles but instead were present mostly as metal oxide inclusions (Figure S1B). Oxygen did not change in the spot analysis indicating that iron and titanium were present as the corresponding oxides and/or oxyhydroxides.

Supplemental Material, Table S1. EDS and XPS elemental characterization of volcanic ash samples (values are in atomic percent)

Elements	O	Na	Mg	Al	Si	K	Ca	Ti	Fe
300x700 mm scan[a]									
Conc.	60.7	3.5	1.6	6.3	19.6	1	2.6	0.7	4
St Dev	0.1	0.1	0	0.1	0.1	0	0.1	0	0.2
Spot scans[b]									
Spot 1	67	4.5	1.1	5.9	17.4	0.8	1.4	0	2
Spot 2	66.7	2.1	2.5	2.2	1.7	0	0	4.3	20.3
XPS									
Conc.	62.2	2.7	2.3	7.1	17.9	0.6	2.8	0.3	4.2

[a] Three measurement average

[b] Spot scan in Figure 1

Supplemental Material, Table S2. ICP-MS elemental composition (mg/kg) of total (unsieved) ash, <20 μm fraction, and leachates.

	Ash analysis			Leachate Analysis			
**Element**	Total ash, Literature values[a]	Total ash, this study mean ± SD[b] (n=3)	<20 um ash (this study)	Total ash, Literature values[a]	Total ash H_2O leaching, this study	Total ash acid leaching, this study	<20 um ash, acid leaching, this study
**Si**	274108	-	-	-	-	-	-
**Ti**	9529	-	12410	0.14	0.18	2.3	-
**Al**	77999	-	-	0.6	81	100	862
**Fe**	74679	-	-	0.8	1.8	33	908
**Mg**	15014	-	-	13.5	-	-	362
**Mn**	1916	-	2045	0.34	3.2	4.6	186
**Ca**	35661	-	-	97	-	-	9088
**Na**	39427	-	-	685	-	-	1021
**K**	16131	-	-	29	-	-	226
**P**	1919	-	-	0.3	-	-	295
**Li**	-	14 ± 1	16	0.19	0.12	0.13	-
**Be**	3.5	-	4	-	0.003	0.011	-
**Sc**	16	18 ± 1	20	0.009	-	-	-
**V**	83	79 ± 1	110	0.1	0.1	0.1	1.8
**Cr**	54	50 ± 27	18	-	0.003	0.021	-
**Co**	22	15 ± 1	18	0.0005	0.026	0.029	0.26
**Ni**	39	31 ± 14	17	0.004	0.098	0.12	0.12
**Cu**	27	28 ± 2	43	0.005	0.077	0.54	2.4
**Zn**	161	152 ± 11	153	0.037	13.4	15.7	7.6
**Ga**	27	29 ± 1	34	0.007	-	-	-
**As**	-	-	-	0.006	0.06	0.18	0.88
**Rb**	36	39 ± 2	44	0.026	0.05	0.07	-
**Sr**	310	323 ± 16	354	0.22	1.2	1.8	14
**Y**	63	65 ± 1	77	-	-	-	-
**Zr**	479	486 ± 20	554	0.004	-	-	-
**Nb**	50	61 ± 2	71	0.0005	-	-	-
**Mo**	3.9	-	5.5	0.05	0.04	0.03	-
**Sn**	4.3	-	4.5	-	-	-	-
**Cs**	0.5	0.47 ± 0.02	0.55	0.00006	0.0003	0.0007	-
**Ba**	421	434 ± 13	459	0.002	0.25	1.2	3.6
**La**	46	53.0 ± 1.7	63.6	0.0004	0.004	0.28	-
**Ce**	107	120 ± 6	141	0.0009	0.01	0.59	-
**Pr**	13.4	15.1 ± 0.4	18.1	0.0001	-	-	-
**Nd**	56	62.7 ± 1.4	74.9	0.0005	-	-	-
**Sm**	12.6	14.1 ± 0.4	16.8	0.0002	-	-	-
**Eu**	4.1	4.58 ± 0.14	5.13	0.00004	-	-	-
**Gd**	12.1	13.6 ± 0.3	16.6	0.0003	-	-	-
**Tb**	1.9	2.17 ± 0.05	2.56	0.00003	-	-	-
**Dy**	10.9	12.6 ± 0.2	14.8	0.0001	-	-	-
**Ho**	2.1	2.43 ± 0.05	2.84	0.00003	-	-	-
**Er**	5.6	6.18 ± 0.13	7.68	0.00007	-	-	-
**Yb**	5.3	5.79 ± 0.14	6.67	0.00006	0.0005	0.02	-
**Lu**	0.8	0.85 ± 0.02	0.96	0.00001	-	-	-
**Hf**	10.7	11.7 ± 0.5	13.4	0.0001	-	-	-
**Ta**	3.9	3.8 ± 0.2	4.2	0.00007	-	-	-
**Pb**	4.2	4.9 ± 0.7	5.2	0.0004	0.0016	0.06	-
**Th**	5.6	5.6 ± 0.3	6.4	0.00004	-	-	-
**U**	1.8	1.84 ± 0.08	2.1	0.001	0.0004	0.006	-

a Borisova et al. 2012; Sigmarsson et al. 2011
b Data represent the mean ± 1 s.d. for analyses of three separate digestions of ash

Supplemental Material, Table S3. ICP-MS element data on standard reference materials to assess data quality

	Ash analysis				Leachate analysis			
**Element**	**BIR-1** measured µg/g	**BIR-1** expected µg/g	**ATHO-G** measured µg/g	**ATHO-G** expected µg/g	**SLRS-5** measured ng/ml	**SLRS-5** expected ng/ml	**NIST1640a** measured ng/ml	**NIST1640a** expected ng/ml
**Li**	3.3	3.2	29.3	28.6	0.5	0.5	0.5	0.4
**Be**	0.09	0.12	3.5	3.2	0.005	0.005	3.1	3.0
**Sc**	42	43	5	5	7.0	7.5	317	303
**Ti**	5557	5600	1421	1529	4630	5380	2853	3112
**V**	321	319	3	4	2585	2540	1080	1050
**Cr**	377	391	5	6	48	50	53	53
**Mn**	1313	1363	845	821	1649	1881	3974	
**Co**	52	52	1	2	8	13	14	
**Ni**	168	166	6	13	816		575	575
**Cu**	115	119	12	19	10614	10500	6111	5570
**Zn**	70	72	124	141	2.6	2.3	1	
**Ga**	16	15	23	25	0.41	0.32	14	15
**Rb**	0.2	0.2	65	65	0.28	0.21	37	41
**Sr**	103	109	95	94	4.2	4.3	39	40
**Y**	16	15.6	103	95	106	91	50	37
**Zr**	14	14	532	512	0.05	0.05	18	20
**Nb**	0.58	0.55	57	62	0.66	0.48	25	
**Mo**	0.06	0.07	4.2	4.8	17.2	17.4	80	86
**Sn**	1.2	0.6	5.9	5.4	1.04	0.85	56	56
**Cs**	0.01	0.01	0.72	1.08	0.48	0.41	7.6	8.1
**Ba**	6.43	7.14	576	547	0.7		22	20
**La**	0.6	0.615	60.3	55.6	1.1	1.2	1.1	1.2
**Ce**	1.87	1.92	132	121	50	54	118	126
**Pr**	0.37	0.37	16.3	14.6	0.33	0.5	42	46
**Nd**	2.36	2.38	65.6	60.9	0.02		7.2	8.1
**Sm**	1.09	1.12	15.3	14.2	0.009	0.006	3.9	4.0
**Eu**	0.53	0.53	3.00	2.76	0.32	0.30	4.9	5.1
**Gd**	1.83	1.87	16.1	15.3	0.004		0.024	
**Tb**	0.36	0.36	2.79	2.51	13.5	14.0	144	152
**Dy**	2.54	2.51	17.6	16.2	0.18	0.20	0.01	
**Ho**	0.57	0.56	3.70	3.43	0.25	0.24	0.013	
**Er**	1.61	1.66	10.6	10.3	0.009	0.009	0.02	
**Yb**	1.63	1.65	10.9	10.5	0.003	0.004	1.5	1.6
**Lu**	0.25	0.25	1.62	1.54	0.07	0.08	11	12
**Hf**	0.62	0.58	14.5	13.7	0.08	0.10	23.6	25.4
**Ta**	0.06	0.04	3.7	3.9				
**Pb**	3.3	3.1	6.38	5.67				
**Th**	0.04	0.03	7.64	7.40				
**U**	0.01	0.01	2.33	2.37				

BIR-1: Iceland basalt rock reference material (source: United States Geological Survey).

expected values from GEOREM database (http://georem.mpch-mainz.gwdg.de: accessed 24th Feb 2013).

ATHO: Iceland rhyolite rock reference material (source: Max Planck Institute, Mainz, Germany).

expected values from GEOREM database (http://georem.mpch-mainz.gwdg.de: accessed 24th Feb 2013).

SLRS-5: river water reference material (source: National Research Council Canada).

expected values from NRCC certificate and Heimburger et al. (2013).

NIST1640a: natural water reference material (source: National Institute of Standards and Technology).

expected values from NIST certificate.

References

Borisova AY, Toutain JP, Stefansson A, Gouy S, de Parseval P. 2012. Processes controlling the 2010 Eyjafjallajökull explosive eruption. Journal of Geophysical Research – Solid Earth 117:B05202, doi:10.1029/2012JB009213.

Heimburger A, Tharaud M, Monna F, Losno R, Desboefs K, Nguyen EB. 2013. SLRS-5 elemental concentrations of thirty-three uncertified elements deduced from SLRS-5 ⁄ SLRS-4 ratios. Geostandards and Geoanalytical Research, in press. doi: 10.1111/j.1751-908X.2012.00185.x

Sigmarsson O, Vlastelic I, Andreasen R, Bindeman I, Devidal J-L, Moune S, et al. 2011. Remobilization of silicic intrusion by mafic magmas during the 2010 Eyjafjallajökull eruption. Solid Earth 2:271-281. doi:10.5194/se-2-271-2011.
